# The Persistence of Silodosin Monotherapy and the Reasons for Withdrawal from Treatment of Previously Untreated Japanese Patients with Lower Urinary Tract Symptoms Suggestive of Benign Prostatic Hyperplasia

**DOI:** 10.1155/2017/4842025

**Published:** 2017-06-13

**Authors:** Yoshinori Tanaka, Yasushi Tanuma, Naoya Masumori

**Affiliations:** ^1^Division of Urology, Hokkaido Prefectural Esashi Hospital, Esashi, Japan; ^2^Division of Urology, Hokkaido Social Welfare Association Hakodate Hospital, Hakodate, Japan; ^3^Department of Urology, Sapporo Medical University School of Medicine, Sapporo, Japan

## Abstract

**Objectives:**

The persistence of silodosin and the reasons for withdrawal from treatment of previously untreated Japanese patients with lower urinary tract symptoms suggestive of benign prostatic hyperplasia (LUTS/BPH) were evaluated in real-life clinical practice.

**Methods:**

A total of 81 previously untreated Japanese patients diagnosed with LUTS/BPH were treated with silodosin monotherapy and prospectively followed for 4 years. The persistence rate was estimated using the Kaplan-Meier method. If silodosin had to be terminated or a patient did not come to the hospital, the reason was determined.

**Results:**

The 6-month, 1-year, 2-year, 3-year, and 4-year persistence rates were 63.0%, 56.8%, 50.6%, 44.4%, and 35.8%, respectively. The most frequent reason (22.2%) for withdrawal was symptom resolution. After silodosin treatment, the international prostate symptom score and the quality of life index were significantly improved and maintained for 4 years.

**Conclusions:**

35.8% of previously untreated Japanese patients continued silodosin for 4 years. Many patients terminated silodosin for various reasons, the most frequent of which was symptom resolution. The effects of silodosin were maintained when the patients continued treatment.

**Trial Registration:**

This study was approved by the institutional review board of Hokkaido Prefectural Esashi Hospital (number 2007-2) and was registered in a public trial registry (UMIN000026910).

## 1. Introduction

The lower urinary tract symptom suggestive of benign prostatic hyperplasia (LUTS/BPH) is commonly observed in elderly men. The prevalence of patients with LUTS/BPH in Japan ranges from 2% in those 40 to 49 years old to 12% in those 70 to 79 years old [1]. The etiology of BPH is consistent with bladder outlet obstruction (BOO) due to not only increased volume of the prostate but also increased tone of the prostatic smooth muscle [2]. Thus, 5*α*-reductase inhibitors and *α*1-adrenoceptor antagonists are used to treat LUTS/BPH. *α*1-Adrenoceptor antagonists are recommended as one of the first-line medical treatments for LUTS/BPH in the Japanese clinical guideline for BPH [3].

Silodosin is a highly selective *α*1A-adrenoceptor antagonist synthesized in Japan. In vitro, its *α*1A-to-*α*1B binding ratio is extremely high [4]. In vivo, silodosin has good uroselectivity in rats and dogs compared to tamsulosin and prazosin [5, 6]. Randomized, double-blind, placebo-controlled clinical studies of silodosin have demonstrated its excellent efficacy and safety for patients with LUTS/BPH [7–10]. A long-term clinical trial in Japanese men with LUTS/BPH showed that the efficacy of silodosin was maintained for 52 weeks [11].

In that trial [11], 71.4% of the patients could continue taking silodosin for 52 weeks. However, in the real-life clinical retrospective study of Furuya et al., the continuance rate of silodosin for one year in Japanese patients with LUTS/BPH was only 12.0% [12]. Yamanishi et al. prospectively investigated the continuance rate of silodosin monotherapy for the treatment of LUTS/BPH in real-life practice for more than 6 years [13]. These two studies also examined the reasons for withdrawal. However, one-quarter of the reasons for withdrawal were not clear because these patients did not come back to the hospital. Masumori et al. prospectively investigated the persistence of tamsulosin and naftopidil for the treatment of LUTS/BPH and reported the reasons for withdrawal, including those of the patients who did not come back to the hospital [14, 15].

In this study, we evaluated the persistence of silodosin monotherapy among previously untreated Japanese patients with LUTS/BPH and the reasons for withdrawal from it, including those of the patients who did not come back to the hospital.

## 2. Methods

The present prospective study was carried out at Hokkaido Prefectural Esashi Hospital in Hokkaido, Japan, in real-life practice. Previously untreated all Japanese patients aged 50 years or older who visited the hospital for lower urinary tract symptoms (LUTS) and were clinically diagnosed with BPH and agree to participate to this study between May 2007 and June 2009 were included. Patients with a urinary tract infection, acute urinary retention, prostate cancer or neurogenic bladder, the use of *α*1-adrenoceptor antagonist or antiandrogen, and a history of prostatic surgery were excluded from this study. This study was approved by the institutional review board of Hokkaido Prefectural Esashi Hospital (number 2007-2). All patients were informed about the risks and benefits and agreed to participate in this study. All patients provided a history, and physical examinations included digital rectal examination, urinalysis, and serum prostate specific antigen (PSA) determination to screen for prostate cancer. Patients suspected of having prostate cancer underwent a needle biopsy of the prostate and were determined to be cancer-free. The international prostate symptom score (IPSS) and the quality of life (QOL) index were determined using a self-administered questionnaire [16]. The prostate volume (PV) was determined by transrectal ultrasound (TITAN®, SonoSite Inc., Bothell, USA). Uroflowmetry (UFM) was done (UM-100, TOTO Ltd., Kitakyusyu, Japan) to evaluate the maximum flow rate (*Q*_max_). One hundred and fifty ml of the minimum voided volume was required, if possible. The postvoid residual volume (PVR) was measured from a single UFM by transabdominal ultrasound (BVI 6100, Verathon Inc., Bothell, USA). Since 5*α*-reductase inhibitors have been approved in Japan since 2009, all patients enrolled between 2007 and 2009 in this study were treated with silodosin monotherapy (4 mg) twice daily as its routine prescription in Japan. The scheduled number of the enrolled patients was 100 according to previous similar studies [14, 15]. Most patients were prescribed silodosin at one- or two-months intervals. The patients were prospectively evaluated according to routine schedule by IPSS and UFM at 3, 6, 12, 18, 24, 30, 36, 42, and 48 months after the treatment. The study has ended in 2013.

Persistence of medication use was measured by the number of months between the first dispense date and the expected end date of the last refill. The persistence rate was estimated by the Kaplan-Meier method with the use of computer software (JMP®, SAS Institute Inc., Cary, USA). Discontinuation was the sum of for-cause termination and loss to follow-up. If silodosin had to be terminated during follow-up (for-cause termination), the reason was determined (adverse effects, lack of efficacy, symptom resolution, etc.). If a patient did not come back to the hospital to receive a prescription (loss to follow-up), the reason for not coming was inquired about by telephone.

In the post hoc analysis, we divided the patients into three subgroups. Those who continued the silodosin treatment for over the 4 years were defined as the continuing group. Those who had acute urinary retention, conversion to prostatic surgery, conversion to another *α*1-adrenoceptor antagonist, add-on of antiandrogen or 5*α*-reductase inhibitors, and discontinuation of silodosin treatment for lack of efficacy were defined as the treatment-failure group. Patients who terminated medication because of symptomatic improvement or did not come back to the hospital because of symptom resolution were defined as the symptom-resolution group. It was recorded if a patient of the symptom-resolution group revisited the hospital and received retreatment with silodosin for the deterioration of LUTS during follow-up (for 4 years after administration).

All values in the table are expressed as means and standard deviations (SD). The differences of the mean values of clinical parameters between pre- and posttreatment were analyzed using the paired Student's *t*-test with Bonferroni correction. The intergroup differences were analyzed using the unpaired Student's *t*-test. *P* values of <0.05 were regarded as statistically significant. Statistics were calculated using computer software (JMP, SAS Institute Inc., Cary, USA).

## 3. Results

A total of 81 patients with LUTS/BPH, aged 73.8 ± 7.3 years (range 59–89), were analyzed. Although the number did not reach to the schedule, the enrollment was closed due to insufficient number of male outpatients with LUTS. Patient characteristics at baseline were summarized in Table 1. Forty patients (49.4%) had a PV of 35 ml or more. Seventy-three patients (90.1%) had moderate or severe symptoms (IPSS > 7) and 76 patients (93.8%) impaired QOL (the QOL index > 2). Sixty-four patients (79.0%) had *Q*_max_ of less than 15 ml/s and 34 patients (42.0%) had a PVR of 50 ml or more.

**Figure 1:**
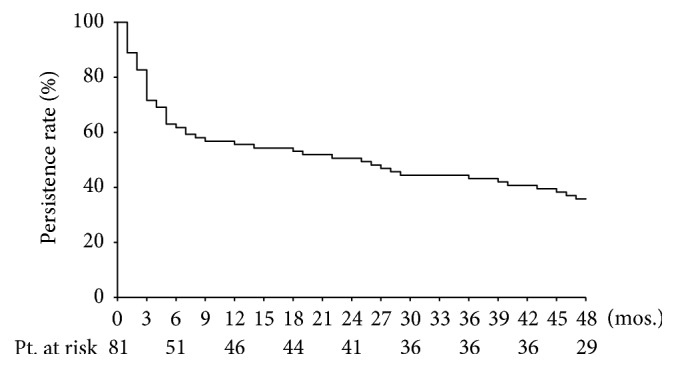
Kaplan-Meier plots of the persistence rates of silodosin treatment for 4 years of follow-up. The 6-month, 1-year, 2-year, 3-year, and 4-year persistence rates were 63.0%, 56.8%, 50.6%, 44.4%, and 35.8%, respectively.

**Figure 2:**
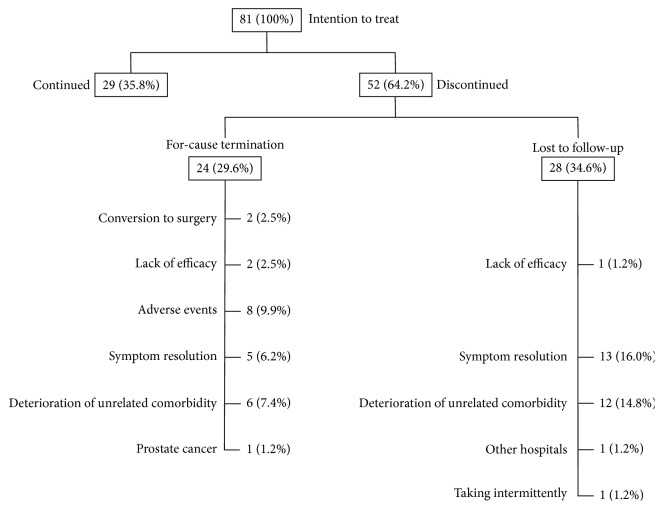
Outcomes of 81 patients at 4 years after administration of silodosin.

Figure 1 shows the Kaplan-Meier plots of the persistence rates for silodosin treatment over the 4 years. The 6-month, 1-year, 2-year, 3-year, and 4-year persistence rates of the 81 patients treated with silodosin were 63.0%, 56.8%, 50.6%, 44.4%, and 35.8%, respectively. In the first 6 months, the persistence rate fell sharply, after which the slope of the graph became more gradual. Finally, 52 patients (64.2%) discontinued silodosin treatment because of for-cause termination in 24 (29.6%) and loss to follow-up in 28 (34.6%) (Figure 2).

Of the 24 patients with for-cause termination, 8 patients (9.9%) terminated the medication because of adverse events (four cases of vertigo, three of ejaculation disorders, and one of urgency). After termination of silodosin treatment, all symptoms of adverse events were immediately improved. Five patients hoped to terminate medication because of symptom resolution, 6 patients gave up silodosin treatment due to worsening or development of comorbidity unrelated to BPH, 2 had conversion to surgical treatment, and 2 hoped to terminate medication because of lack of efficacy. In one patient, prostate cancer was detected during follow-up. Three patients required add-on of antimuscarinic agents during follow-up, but two patients terminated them because of lack of efficacy. No patient needed to add antiandrogen or 5*α*-reductase inhibitors and none was converted to another *α*1-adrenoceptor antagonist. There was no patient with acute urinary retention.

Of the 28 patients lost to follow-up, 13 terminated the medication themselves and stopped coming to the hospital because of symptom resolution and 12 were not able to come to our hospital because of deterioration of a comorbidity unrelated to BPH. One patient terminated medication himself because of lack of efficacy, one went to another hospital, and one intermittently used silodosin.

Finally, 18 patients (22.2%) terminated the medication because of symptom resolution (symptom-resolution group). Of the 18, 5 revisited the hospital and were retreated with silodosin because of the deterioration of LUTS during follow-up (at an average 10.6 months after termination). Another 18 patients (22.2%) gave up the medication due to worsening or development of a comorbidity unrelated to BPH (five of cancers unrelated to urology, four of dementia, two of aortic aneurysm, two of pneumonia, one of depression, one of emphysema, one of liver abscess, one of gastrointestinal hemorrhage, and one of glaucoma). Eight patients (9.9%) terminated it because of adverse events, 3 (3.7%) did so because of lack of efficacy, and 2 cases (2.5%) were converted to surgery. Therefore, five patients (6.2%) were defined as treatment-failure group.

The baseline characteristics of patients in the symptom-resolution group, continuing group, and treatment-failure group are summarized in Table 2. In the symptom-resolution group, baseline PV was smaller and PSA was lower, but there was no significant difference. Baseline parameters did not significantly differ between the symptom-resolution group and the continuing group, the symptom-resolution group and treatment-failure group, and the continuing group and the treatment-failure group.

After silodosin treatment, the QOL index of the patients in the continuing group was significantly improved and maintained for 4 years (Table 3). The storage symptom score (IPSS 2 + 4 + 7), voiding symptom score (IPSS 3 + 5 + 6), total IPSS, and the average flow rate (*Q*_aver_) were also significantly improved (Table 4). There was no significant change of voided volume (*V*_V_), *Q*_max_, or PVR (Table 4).

## 4. Discussion

Since the medical treatments for chronic diseases such as LUTS/BPH usually have to be continued, the treatment efficacy depends on the persistence of use of the prescribed medicines. Therefore, the continuation rates with *α*1-adrenoceptor antagonists for LUTS/BPH have been prospectively studied. Masumori et al. reported that the continuation rates for tamsulosin at 5 years and for naftopidil at 3 years were 30.4% and 21.4%, respectively [14, 15]. Yamanishi et al. reported that the continuation rate for silodosin at 6 years was 25% [13]. In the present study, the continuation rate for silodosin at 4 years was 35.8%. Thus the four studies showed similar results.In the three previous reports [13–15] and the present study (Tables 3 and 4), LUTS of patients who continued *α*1-adrenoceptor antagonists achieved significant improvement that was maintained, although the placebo effects may be added on the results for uncontrolled trials. Consequently, the *α*1-adrenoceptor antagonists were efficacious for those patients who continued using them, but their continuation rates were low.

To clarify the true efficacy of *α*1-adrenoceptor antagonists, whose continuation rates are low, investigation of the reasons for withdrawal is necessary. In the study of Masumori et al., which included patients who did not come back to the hospital, the reasons for discontinuation of tamsulosin were improvement of LUTS (18.8%), no change/becoming worse (13.4%), conversion to surgery (10.7%), and adverse events (3.6%) [14], whereas those for naftopidil were improvement of LUTS (28.2%), conversion to other *α*1-adrenoceptor antagonists (17.9%), and adverse events (5.1%) [15]. The most frequent reason for discontinuation was not a lack of efficacy, but improvement of LUTS. Since the patients continued treatment with improved LUTS and terminated medicines because of improvement of LUTS, tamsulosin and naftopidil were efficacious for half [14, 15]. However, the reasons for discontinuation of silodosin reported by Yamanishi et al. were conversion to surgery (20.2%), side effects (8.7%), and satisfaction (4.8%) [13]. There was a discrepancy, because it was not clear why their patients did not come back to the hospital. In the present study, which included patients who did not come back to the hospital, the most frequent reason for discontinuation of silodosin was also symptom resolution (22.2%). Consequently, silodosin also had efficacy for more than half of the patients.

To clarify the characteristics of the patients who terminated silodosin because of improvement of LUTS, we compared the baseline parameters of the symptom-resolution group and other groups. PV and PSA were smaller and lower, respectively, but there was no significant difference. This might have been due to the small sample size. However, the patients who terminated tamsulosin or naftopidil because of improvement of LUTS were younger and had a lower PSA level (tamsulosin) at baseline or had higher Qmax and smaller PV (naftopidil) at baseline [14, 15]. Further larger-scale prospective multicenter studies are required to clarify this issue.

It has been reported that 26% of the patients who discontinued naftopidil needed retreatment with *α*1-adrenoceptor antagonists and/or surgery during follow-up [15]. In the present study, five (27.8%) of the 18 patients who terminated silodosin because of symptom resolution revisited the hospital and received retreatment with silodosin because of the deterioration of LUTS during follow-up. Yokoyama et al. also reported that 30% of patients required retreatment within 12 months after discontinuation of *α*1-adrenoceptor antagonist [17]. Thus, after discontinuation of *α*1-adrenoceptor antagonist because of symptom resolution, deterioration of LUTS and the need for retreatment are not many.

The generalizability of the results of the present study was limited to some extent for single-center study. The mean age of the present study was higher than the three previous multicenter reports [13–15]. This finding might have been due to regional bias. The rate of conversion to surgery was markedly lower than in other reports. This finding might have been due to bias resulting from the treatment policy in this single-center study. Therefore we could not determine risk factors for treatment failure. However, Yamanishi et al. indicated that patients who converted to surgery had larger PV, a higher QOL index, and a higher PSA level than those who continued silodosin [13]. Masumori et al. indicated that PV and PVR at baseline were predictors for treatment failure of tamsulosin [14], whereas age, PV, and PSA at baseline were predictors for treatment failure of naftopidil [15]. Therefore, large PV at baseline is risk factor for treatment failure of *α*1-adrenoceptor antagonists. Roehrborn et al. reported that 11.9% of patients treated with tamsulosin monotherapy had acute urinary retention or BPH-related surgery within 4 years, whereas only 4.2% of those with combination therapy using dutasteride and tamsulosin did [18]. For patients with large PV, PV reduction using 5*α*-reductase inhibitors may be necessary for a good long-term outcome.

In phase III trials, the most frequent and important adverse event was reported to be ejaculatory dysfunction (14–28.1%) [7–10]. However, in the present study only 9.9% of patients terminated silodosin because of adverse events (four cases of vertigo, three of ejaculation disorders, and one of urgency). Although many patients in this study were too old for sexual activity, the rate of sexual dysfunction for adverse events was markedly low. Since we investigated the persistence of silodosin and the reasons for withdrawal in real-life practice, we did not ask about sexual activity at baseline and did not systematically check for sexual dysfunction side effects and herein report only adverse events that caused withdrawal.

## 5. Conclusions

35.8% of patients continued silodosin for 4 years. Many patients terminated silodosin for various reasons, the most frequent of which was symptom resolution. The effects of silodosin were maintained when the patients continued treatment.

## Figures and Tables

**Table 1:** Baseline characteristics of study patients (*n* = 81).

Parameters	Mean	(SD)
Age (years)	73.8	(7.3)
PV (ml)	38.9	(20.2)
PSA (ng/ml)	3.8	(7.5)
IPSS		
Total score	15.2	(6.9)
Storage subscore	6.9	(3.8)
Voiding subscore	6.1	(4.6)
QOL index	4.4	(1.3)
UFM		
*V*_V_ (ml)	184.7	(115.9)
*Q*_max_ (ml/s)	11.4	(5.2)
*Q*_aver_ (ml/s)	5.9	(3.1)
PVR (ml)	57.7	(66.5)

**Table 2:** Comparison of baseline characteristics among three subgroups.

Parameters	Symptom-resolution group (*n* = 18)		Continuing group (*n* = 29)		Treatment-failure group (*n* = 5)	
	Mean	(SD)	Mean	(SD)	Mean	(SD)
Age (years)	75.0	(8.1)	73.8	(6.8)	70.4	(6.8)
PV (ml)	35.3	(13.3)	42.4	(16.3)	40.6	(18.7)
PSA (ng/ml)	2.5	(2.0)	4.7	(10.4)	3.2	(2.4)
IPSS	15.4	(6.8)	15.4	(6.8)	13.8	(4.2)
QOL index	4.2	(1.4)	4.2	(1.4)	4.0	(0.7)
*Q*_max_ (ml/s)	11.9	(7.6)	11.7	(3.6)	11.7	(7.2)
PVR (ml)	75.7	(79.7)	60.4	(70.2)	74.4	(110.6)

Baseline parameters did not significantly differ between the symptom-resolution group and the continuing group, the symptom-resolution group and treatment-failure group, and the continuing group and the treatment-failure group.

**Table 3:** Changes of mean IPSS in patients who continued the silodosin treatment (*n* = 29).

Parameters	0 mos.	3 mos.	6 mos.	12 mos.	18 mos.	24 mos.	30 mos.	36 mos.	42 mos.	48 mos.
Total score	15.2	9.4^*∗∗∗*^	8.2^*∗∗∗*^	9.3^*∗∗∗*^	8.8^*∗∗∗*^	9.0^*∗∗∗*^	10.0^*∗∗*^	11.6	10.8^*∗*^	11.1^*∗*^
(SD)	(6.9)	(7.5)	(7.0)	(7.3)	(7.0)	(7.5)	(6.9)	(8.2)	(8.2)	(7.8)
Storage subscore	7.1	4.2^*∗∗∗*^	4.1^*∗∗∗*^	4.4^*∗∗∗*^	4.5^*∗∗∗*^	4.3^*∗∗∗*^	5.1^*∗*^	5.2^*∗*^	5.1^*∗∗*^	5.4
(SD)	(3.5)	(3.0)	(3.3)	(3.3)	(3.0)	(3.1)	(3.6)	(3.9)	(3.6)	(3.8)
Voiding subscore	6.6	4.0	3.3^*∗∗*^	3.6^*∗*^	3.2^*∗∗*^	3.7^*∗∗*^	3.9^*∗*^	5.1	4.3	4.2^*∗*^
(SD)	(4.1)	(4.0)	(3.8)	(4.0)	(3.9)	(3.9)	(3.7)	(4.2)	(4.1)	(3.9)
QOL index	4.8	3.1^*∗∗∗*^	2.6^*∗∗∗*^	2.9^*∗∗∗*^	2.6^*∗∗∗*^	2.6^*∗∗∗*^	2.8^*∗∗∗*^	3.1^*∗∗∗*^	3.0^*∗∗∗*^	2.9^*∗∗∗*^
(SD)	(0.9)	(1.5)	(1.5)	(1.6)	(1.6)	(1.7)	(1.4)	(1.7)	(1.6)	(1.6)

^*∗*^ *P* < 0.05, ^*∗∗*^*P* < 0.01, and ^*∗∗∗*^*P* < 0.001 versus baseline, the paired Student's *t*-test with Bonferroni correction.

**Table 4:** Changes of mean uroflowmetric parameters in patients who continued the silodosin treatment (*n* = 29).

Parameters	0 mos.	3 mos.	6 mos.	12 mos.	18 mos.	24 mos.	30 mos.	36 mos.	42 mos.	48 mos.
*V*_V_ (ml)	181.7	196.6	206.8	209.1	215.3	204.1	218.7	197.4	220.6	209.2
(SD)	(98.1)	(93.5)	(127.0)	(139.9)	(162.3)	(108.9)	(132.8)	(95.8)	(110.3)	(127.1)
*Q*_max_ (ml/s)	11.7	12.4	13.0	13.1	13.2	13.0	12.9	12.7	12.7	12.9
(SD)	(3.6)	(4.6)	(6.1)	(5.8)	(6.5)	(4.3)	(5.7)	(5.8)	(4.4)	(6.5)
*Q*_aver_ (ml/s)	6.1	6.6	7.2	8.0	7.8	8.1^*∗∗*^	8.1^*∗∗*^	7.9^*∗*^	7.9^*∗∗*^	7.8
(SD)	(2.3)	(3.0)	(3.5)	(3.8)	(3.5)	(2.2)	(2.9)	(3.1)	(2.3)	(3.1)
PVR (ml)	60.4	42.6	47.2	46.3	58.1	54.2	62.6	78.7	63.3	72.4
(SD)	(70.2)	(44.0)	(38.9)	(38.2)	(48.8)	(42.3)	(49.0)	(75.6)	(34.7)	(41.5)

^*∗*^ *P* < 0.05, ^*∗∗*^*P* < 0.01, and ^*∗∗∗*^*P* < 0.001 versus baseline, the paired Student's *t*-test with Bonferroni correction.
